# ﻿The early evolution of caddisflies: Milne and Milne revisited

**DOI:** 10.3897/zookeys.1263.148088

**Published:** 2025-12-10

**Authors:** Paul B. Frandsen, Ralph W. Holzenthal

**Affiliations:** 1 Department of Plant and Wildlife Sciences and Bean Life Science Museum, Brigham Young University, Provo, UT, USA Brigham Young University Provo United States of America; 2 Department of Entomology, University of Minnesota, St. Paul, MN, USA University of Minnesota St. Paul United States of America

**Keywords:** Caddisfly, evolution, freshwater, phylogenetics, Trichoptera

## Abstract

In 1939, Margery and Lorus Milne published a creative figure that illustrated the evolutionary history of caddisflies. Here, we pay tribute to that paper by generating a new figure in the style of the original, updated with our most recent knowledge of caddisfly evolution, informed by phylogenomic inference and a statistical treatment of ancestral states. Our analysis infers that the ancestral larval caddisfly was a free-living detritivore, living in flowing water. It spun a cocoon prior to pupation within a dome-shaped pupal shelter. In subsequent lineages, caddisflies evolved a variety of larval construction behaviors, which enabled unprecedented ecological diversification, allowing them to become one of the most diverse lineages of freshwater animals.

## ﻿Introduction

Caddisflies, often appropriately referred to as “nature’s underwater architects”, belong to perhaps the most species-rich and ecologically diverse clade of aquatic insects (Wiggins 2004; Dijkstra et al. 2014; Morse et al. 2019). Since the description of the first caddisflies by Linnaeus, the pattern and mode of their evolutionary and ecological diversification have been the subject of many studies over many decades (Kolenati 1848; Martynov 1924; Ross 1967; Weaver 1984; Wiggins and Wichard 1989; Frania and Wiggins 1997; Kjer et al. 2001, 2016; Ivanov 2002; Holzenthal et al. 2007; Malm et al. 2013; Thomas et al. 2020; Ge et al. 2023, 2024; Frandsen et al. 2024).

**Figure 1.**
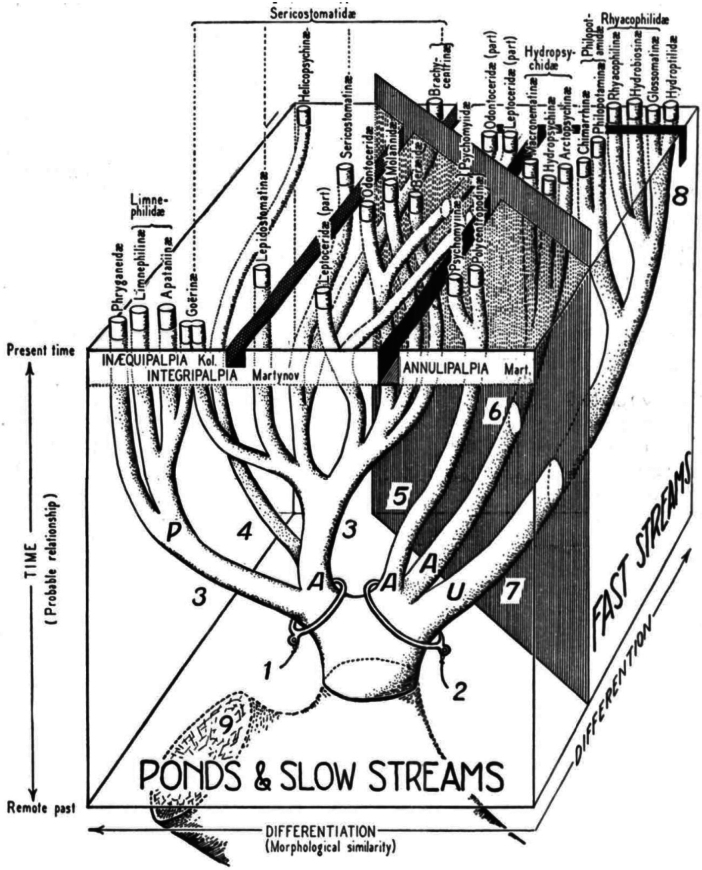
Original figure used with permission from Milne and Milne (1939); copyright Entomological Society of America. Note that the figure legend uses some classification terminology no longer in widespread use. The original figure legend is as follows: 1, Herbivores and scavengers with movable cases. 2, Carnivores building either fixed shelters or none at all. 3, Short cases. 4, Long case coiled in a flat spiral. 5, Fixed silken tubes, often with funnel mouths. 6, Fixed nets and snares. 7, Free-living larvae, never building more than loose silken wefts until preparing to pupate. 8, Case-building in later larval life but prior to preparations for pupation. 9, Lepidopterous stem. A, Ocelli absent. P, Ocelli present. U, Ocelli usually present (absent in some Hydroptilidae). The barrier separating “Ponds and slow streams” from “Fast streams” is largely one of dislodgement or displacement pressure. The separation of “Integripalpia” from “Annulipalpia” on the basis of secondary segmentation of the maxillary palpi seems to have no phylogenetic basis. An angulate trench indicates the limits of Martynov’s suborders. The “Inaequipalpia” of Kolenati are distinct in that the adult maxillary palpi have less segments in the male than in the female, while all other Trichoptera show equal palpi in both sexes and are hence “Aequipalpia.” A Z-shaped ditch separates the suborders of Kolenati.

One of the early works to examine the evolutionary history of caddisflies and, to our knowledge, the first paper to present a detailed figure depicting an evolutionary tree for Trichoptera was authored by Margery and Lorus Milne in 1939 (Milne and Milne 1939). The second figure in their paper (their plate 1) illustrated the evolutionary history of caddisflies in a distinct style from most other evolutionary trees, including a three-dimensional illustration that included not only proposed evolutionary relationships but also hypotheses concerning the evolution of life history traits of caddisflies (Fig. 1). While their work pre-dated both formal, Hennigian systematics, which codified the science of systematics including defining monophyly, and modern statistical methods for inferring ancestral states, the figure still stands as an inspiration to generations of caddisfly workers.

We recently published a phylogenetic tree estimated from phylogenomic data, representing the breadth of caddisfly diversity, including 48/52 families and 172 genera (Frandsen et al. 2024). Given that many of the evolutionary relationships within the caddisfly phylogeny are now resolved with strong support, we aim here to provide the community with an updated illustration in the style of the original Milne and Milne figure, based on the most recent estimates of phylogeny and from a statistical reconstruction of ancestral states. Our aim is also to pay homage to the quality and foresightfulness of the work of the Milnes, not to offer a critique of their original hypotheses.

## ﻿Brief biographical information

Margery Green Milne and Lorus Johnson Milne were consummate scientists, naturalists, and educators. Over the course of their long careers, together they published over 50 books and numerous scientific papers. Today they would be referred to as a “power couple.” Born in Canada in 1910, Lorus went on to receive a Ph.D. from Harvard University in 1936. Margery, a native of the Bronx, New York, received her Ph.D. in 1939 from Radcliffe College. Earlier, the couple met while both were taking a summer course at the Marine Biological Laboratories at Woods Hole, Massachusetts, in 1934. They soon became engaged and married in 1936. The early years of their engagement and marriage were largely spent apart as they were establishing their careers in academia. In 1948 they both received appointments as faculty at the University of New Hampshire. However, Margery eventually had to leave her position as married couples were not allowed to hold tenure track positions in the same department. Undaunted, Margery obtained teaching positions at nearby colleges and universities. They spent the remainder of their careers in Durham, New Hampshire. Lorus died in 1987 at the age 76. Margery lived to be 94, passing away in 2006. An obituary of Lorus was published by Weaver (1988), and a summary of Margery’s life can be found on the University of New Hampshire Library’s web page devoted to her and Lorus’s works (https://library.unh.edu/find/archives/collections/lorus-margery-milne-papers-1924-2005).

Their prolific authorship of both popular and more technical works covered a wide range of topics in biology and natural science, including plants, birds, insects, and marine invertebrates, as well as geology, animal behavior, and evolution, among many others. One needs only browse Amazon or AbeBooks to see their works, many of which are still available. Their studies and papers on Trichoptera were foundational (Table 1). Lorus self-published a series of papers on North American caddisflies in which he provided a key to the known genera and descriptions of many species. In a whimsical manner, he named 22 species of *Rhyacophila* beginning with the letter “v” (e.g., R. vagrita, *R. vetina*, *R. vu*). Margery’s work concentrated on larval biology and case-building. Her paper on the “metamorphotype method” is still used as a primary method in associating adult and larval life stages. It relies on the retrieval and description of larval sclerites from within the pupal chamber of a mature, pharate male pupa. Of course, her most impressive contribution, co-authored with Lorus, is the paper we are showcasing here, especially its amazing figure, which we assume was rendered by Margery.

**Table 1.** Published works on Trichoptera by Margery and Lorus Milne.

Milne LJ (1931) Three new Canadian *Prophryganea* (Phryganeidae, Trichoptera). Canadian Entomologist 63: 228–232.
Milne LJ (1934) Studies in North American Trichoptera, 1. Privately printed.
Milne LJ (1935) Studies in North American Trichoptera, 2. Privately printed.
Milne LJ (1936) Studies in North American Trichoptera, 3. Privately printed.
Milne LJ, Milne MJ (1938) The Arctopsychidae of continental America north of Mexico (Trichoptera). Bulletin of the Brooklin Entomological Society 33(3): 97–110.
Milne LJ, Milne MJ (1944) Caddis flies (Trichoptera) and pitcher plants. Psyche 51(3–4): 179–182.
Milne MJ (1938) Case-building in Trichoptera as an inherited response to oxygen deficiency. Canadian Entomologist 70(9): 177–180.
Milne MJ (1938. The “metamorphotype method” in Trichoptera. Journal of the New York Entomological Society 46: 435–437.
Milne MJ (1939) Immature North American Trichoptera. Psyche 46: 9–19.
Milne MJ, Milne LJ (1939) Evolutionary trends in caddis worm case construction. Annals of the Entomological Society of America 32: 533–542.
Milne MJ, Milne LJ (1940) A new species of *Rhyacophila*, described from metamorphotypes (Rhyacophilidae, Trichoptera). Bulletin of the Brooklin Entomological Society 35: 153 155.

## ﻿Methods

To generate the updated illustration, we used the phylogenetic tree from Frandsen et al. (2024). This tree was generated from a combined dataset of genomes, transcriptomes, and targeted capture data. We then evaluated the original Milne and Milne (1939) figure to understand the ecological traits that they examined. The list of traits included larval construction, pupal case construction, pupal cocoon type, habitat type (lentic vs lotic), and feeding type. We used the results from the ancestral state reconstruction analyses for larval and pupal case construction, pupal cocoon, and habitat type from the Frandsen et al. (2024) study (Suppl. material 2: figs S1–S5). However, it was necessary to perform an additional ancestral state reconstruction for feeding type, as it was not included by Frandsen et al. in their study.

The objective of ancestral state reconstruction is to use traits from extant species within a clade to reconstruct the ancestral patterns of those traits throughout the evolutionary history of that clade. These methods rely on statistical models that assign probabilities of a particular set of character states to ancestral nodes based on the shape of the tree and the distribution of those character states among extant taxa. To reconstruct ancestral states for feeding type, we first assigned each species in the phylogeny to one of three feeding categories: detritivores, herbivores, or predators, using the available literature (e.g. Wiggins 2004) and our own observations (Suppl. material 1). We then estimated ancestral states using the ANCR marginal ancestral reconstruction method in Phytools (Revell 2024). We estimated a mixture model combining estimates from the built-in Phytools equal rates (ER), symmetric (SYM), and all rates differ (ARD) ancestral state reconstruction models. We then used Adobe Illustrator to develop a new illustration, in the style of the original Milne and Milne figure, taking into account the results from the statistical ancestral state reconstruction analysis.

## ﻿Results

**Figure 2.**
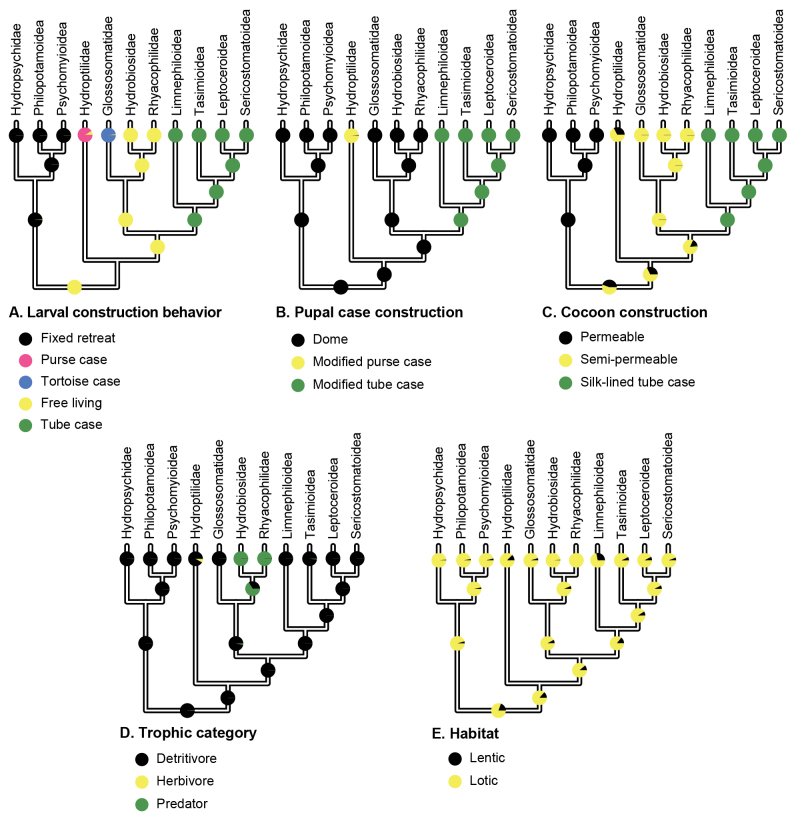
Simplified results from the ancestral state reconstruction analyses. Pie charts indicate the probability of a particular state at a particular node **A–C, E** were estimated in Frandsen et al. (2024)**D** was estimated for this work. In some cases, the probability of a particular state is very small and represented only by a sliver in the pie chart. Full ancestral state reconstructions can be found in the Suppl. material 1.

**Figure 3.**
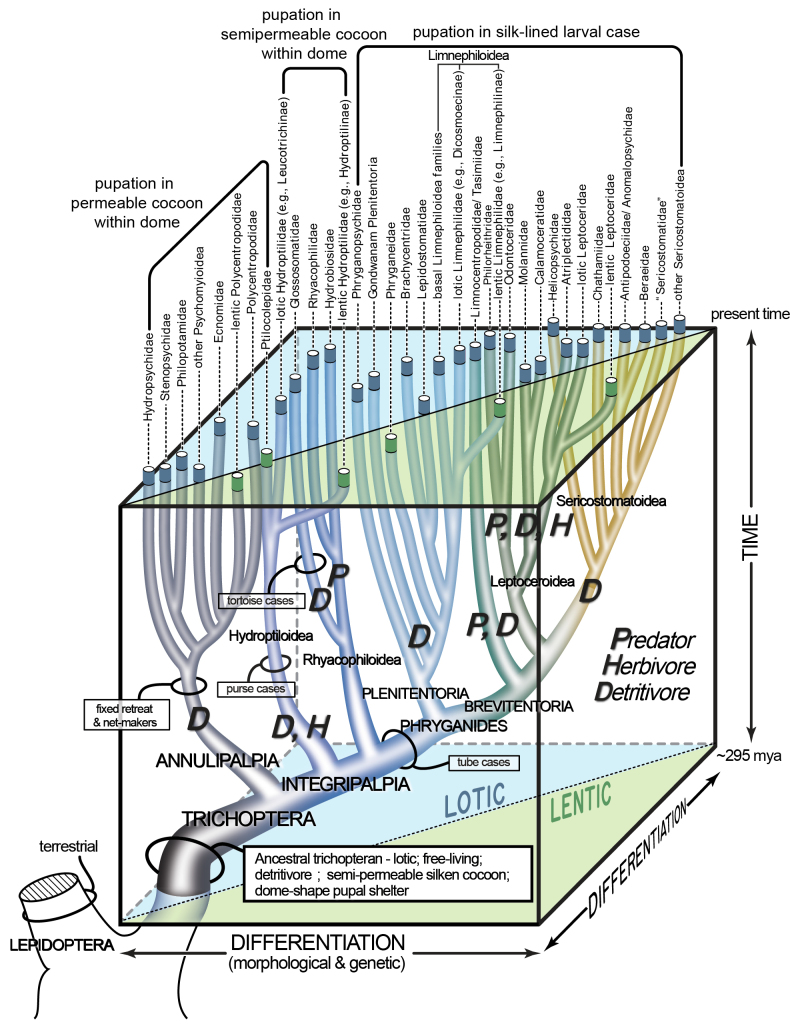
Depiction of the evolution of life history traits in caddisflies, based on the most recent studies of caddisfly evolution and developed in the style of the original figure in Milne and Milne (1939) (Fig. 1). Transitions in larval construction type are shown with black lassos, transitions between habitat types are illustrated with blue (lotic) and green (lentic) branch tips. Feeding type transitions are indicated with P (predator), D (detritivore), and H (herbivore).

Our ancestral state reconstruction of feeding type indicates that the ancestral trichopteran larva was a detritivore. When this information is combined with the results presented by Frandsen et al. (2024), we infer that the ancestral trichopteran inhabited a lotic environment as a free-living, detritivorous larva and later constructed a pupal dome with a cocoon (Fig. 2). Throughout their evolutionary history, lineages of caddisflies made multiple transitions in larval habitat type, feeding type, and case-making behavior. These ancestral states and transitions are shown in detail in our ancestral state reconstructions (Suppl. material 2: figs S1–S5) and illustrated in our new figure (Fig. 3).

## ﻿Discussion

Given their species and ecological diversity, caddisflies have an outsized impact on freshwater environments (Morse et al. 2019). Understanding the evolutionary basis of their diversity has been a key focus of caddisfly research over the last century. The 1939 paper by Margery and Lorus Milne inspired generations of caddisfly workers with its creative depiction of trichopteran evolution. Advances in genomic sequencing and statistical methods for inferring phylogenetic trees and ancestral states have granted us an unprecedented opportunity to reconstruct the evolutionary history of caddisflies.

Recent phylogenomic studies converged on a hypothesis for the early evolution of Trichoptera (Frandsen et al. 2024; Ge et al. 2024), allowing for the interpretation of the timing and evolution of life history traits. Caddisflies comprise two suborders, Annulipalpia and Integripalpia. Evidence suggests that the ancestral trichopteran larva was a detritovore that inhabited flowing freshwater. It is possible that the ancestral trichopteran first inhabited marginal areas of streams and later ventured into faster flows to exploit food resources and higher oxygen concentrations. Before pupation the larva constructed a pupal dome and cocoon out of specialized silk. In the suborder Annulipalpia, the free-living larva began to build a variety of fixed shelters to provide protection or feeding opportunities for the larval stage, some lineages added silken capture nets to facilitate feeding. In rare occasions, lineages within Annulipalpia invaded standing waters (some Polycentropodidae), while most lineages exploited fast-moving streams and rivers. In the suborder, Integripalpia, evolution favored the precocious building of the pupal dome, and three lineages independently began to build portable cases as larvae. These were (1) the microcaddisflies in the families Ptilocolepidae and Hydroptilidae, which only build a portable case in their final instar, (2) the tortoise-case makers in the family Glossosomatidae, and (3) the tube-case makers in the clade Phryganides. The use of a portable case enabled ecological diversification, and several groups invaded lentic habitats, including groups in the families Limnephilidae, Leptoceridae, and Phryganeidae (Suppl. material 2: fig. S2). This transition also often coincided with a shift to herbivory, perhaps driven by the change in food availability in lentic vs lotic substrates (Suppl. material 2: fig. S1). Other groups, such as Rhyacophilidae and Hydrobiosidae, retained their ancestral free-living behavior, but they evolved to become predatory and inhabit fast-flowing water (Suppl. material 2: fig. S2).

While these recent advances in our understanding of the evolution of Trichoptera are exciting, we argue that we should not ignore the insights of past researchers and the hypotheses that they proposed concerning caddisfly natural history. Research indicates that knowledge of natural history is declining (Tewksbury et al. 2014), perhaps partly due to an emphasis on developing technical skills in genomic sequencing and analysis. As advances in these areas make it easier to generate and analyze genomic data, future research advances are likely to emerge from well-formed biological questions, which arise only from a deep knowledge of the natural history of our study organisms. The present study was developed as an homage to the Milnes who presented a compelling and creative illustration depicting the evolution of caddisflies, which was informed by their extensive knowledge of natural history. We hope that our new illustration, presented alongside the classic illustration of Margery and Lorus Milne, will inspire future caddisfly researchers to develop questions that merge natural history with modern analytical approaches to continue to unlock the mysteries behind the evolution of nature’s underwater architects.

## ﻿Conclusion

Modern DNA sequencing and analytical techniques have facilitated the resolution of the evolutionary history of caddisflies. Here, we combined the newest results derived from modern approaches with the creativity of past researchers to illustrate the evolution of caddisflies and their life history traits. Within each suborder, larval silk use diversified, and caddisflies began to build an array of portable cases (within Integripalpia) or fixed retreats (within Annulipalpia). This was accompanied by increased ecological diversification, including a wide array of feeding behaviors, and allowed for multiple transitions into still waters.

## ﻿Additional information

### Author ORCIDs

Paul B. Frandsen https://orcid.org/0000-0002-4801-7579

Ralph W. Holzenthal https://orcid.org/0000-0003-1853-6340

### Data availability

All of the data that support the findings of this study are available in the main text and Supplementary material.

## Supplementary materials

Supplementary material 1 Feeding type designations for ancestral state reconstruction analyses Data type csv Explanation note A CSV containing the character states used for the feeding type ancestral state reconstruction. https://binary.pensoft.net/file/1488051This dataset is made available under the Open Database License (http://opendatacommons.org/licenses/odbl/1.0/). The Open Database License (ODbL) is a license agreement intended to allow users to freely share, modify, and use this Dataset while maintaining this same freedom for others, provided that the original source and author(s) are credited.Paul B. Frandsen, Ralph W. Holzenthal

Supplementary material 2 Results of ancestral state reconstruction analyses Data type pdf Explanation note **fig. S1.** Ancestral state reconstruction using ANCR for caddisfly larval feeding types. **fig. S2.** Ancestral state reconstruction for caddisfly larval habitat type (lentic vs. lotic). **fig. S3.** Ancestral state reconstruction for caddisfly pupal dome construction. **fig. S4.** Ancestral state reconstruction for caddisfly pupal cocoon construction. **fig. S5.** Ancestral state reconstruction for caddisfly larval case-making behavior. https://binary.pensoft.net/file/1488052This dataset is made available under the Open Database License (http://opendatacommons.org/licenses/odbl/1.0/). The Open Database License (ODbL) is a license agreement intended to allow users to freely share, modify, and use this Dataset while maintaining this same freedom for others, provided that the original source and author(s) are credited.Paul B. Frandsen, Ralph W. Holzenthal
