# Supplementary material for: ﻿The early evolution of caddisflies: Milne and Milne revisited
Source: Zookeys. 2025 Dec 10;1263:37–46. doi: 10.3897/zookeys.1263.148088 (PMC12712623; doi:10.3897/zookeys.1263.148088)

Supplemental Information for “The early evolution of caddisflies: Milne and Milne revisited”

Figure S1. Ancestral state reconstruction using ANCR for caddisfly larval feeding types

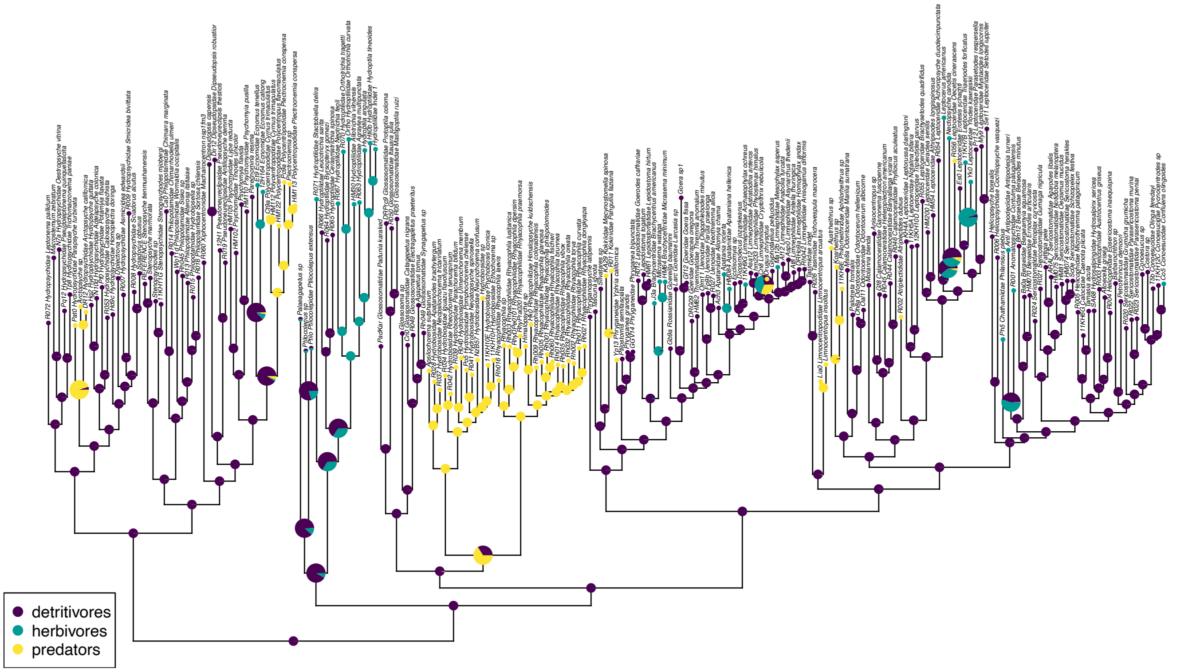

Figure S2. Ancestral state reconstruction for caddisfly larval habitat type (lentic vs. lotic)

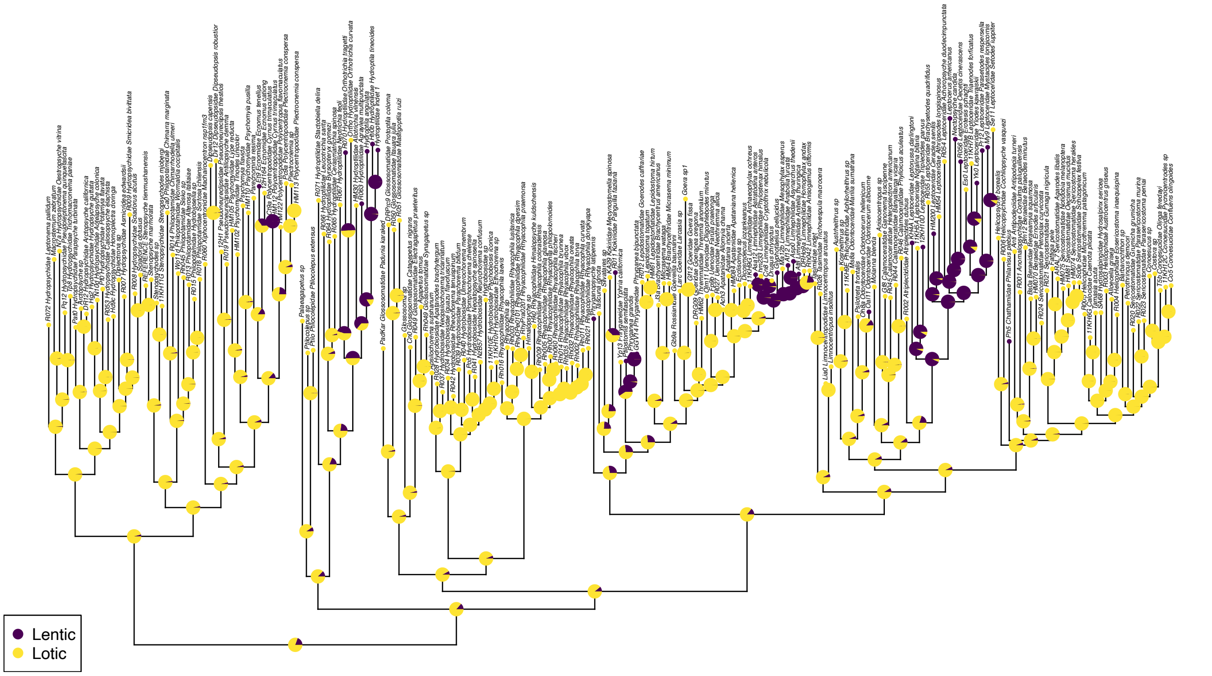

Figure S3. Ancestral state reconstruction for caddisfly pupal dome construction

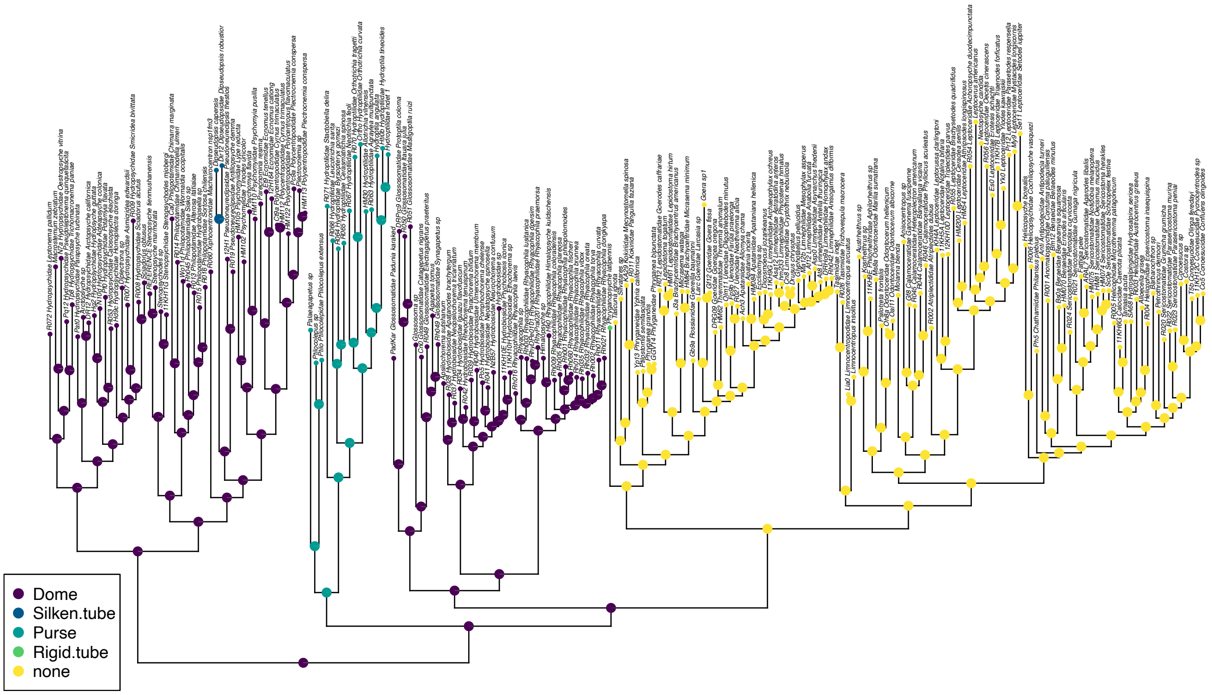

Figure S4. Ancestral state reconstruction for caddisfly pupal cocoon construction

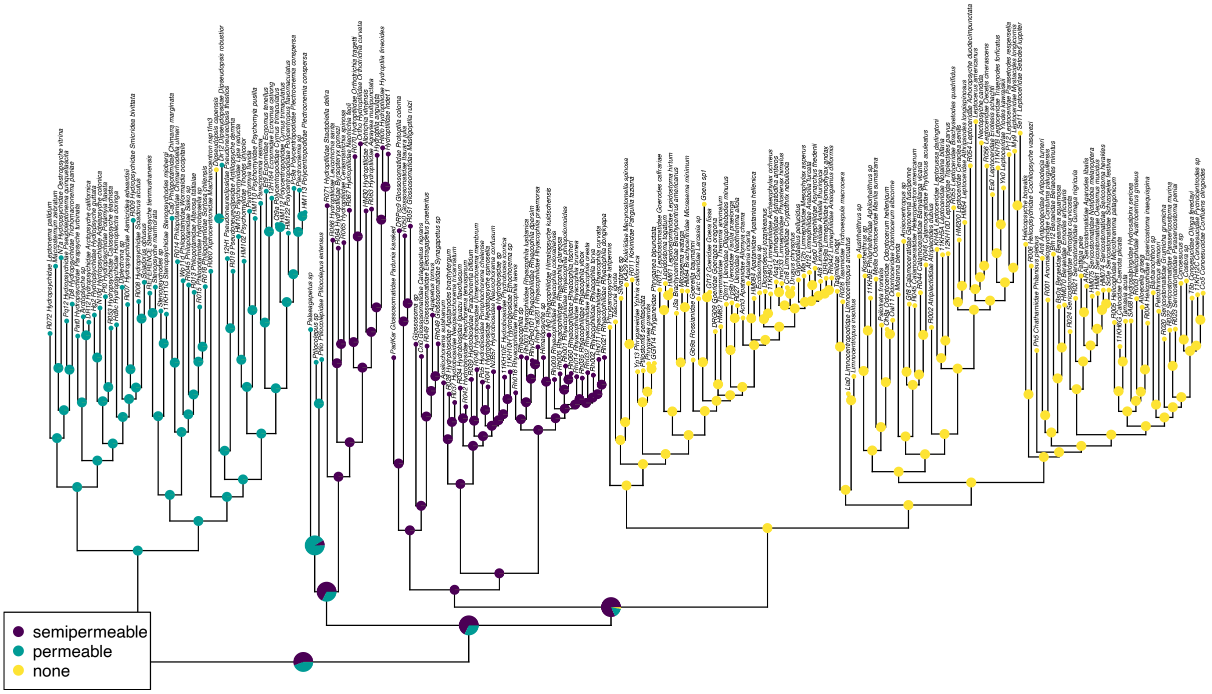

Figure S5. Ancestral state reconstruction for caddisfly larval case-making behavior

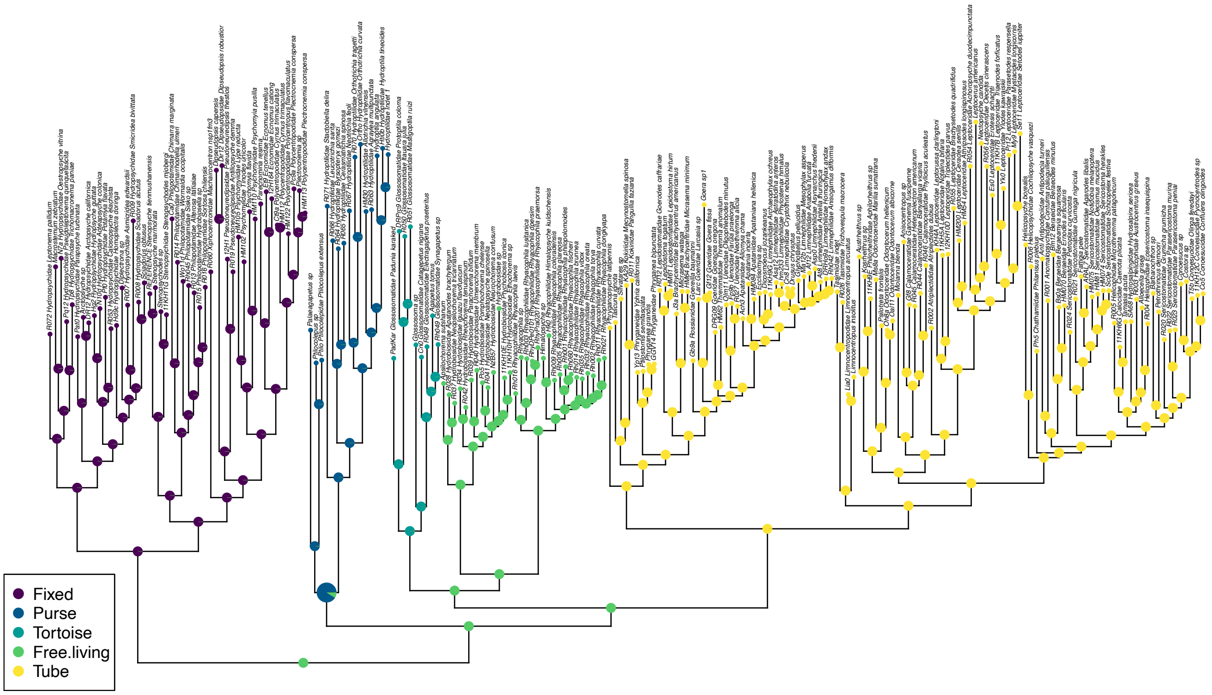

Supplement: Supplementary material 2 — Results of ancestral state reconstruction analyses [file zookeys-1263-037_article-148088__-s002.pdf]
